# A case of chronic myelogenous leukemia with the T315I mutation who progressed to myeloid blast phase and was successfully treated with asciminib

**DOI:** 10.1002/ccr3.6478

**Published:** 2022-11-06

**Authors:** Sarah Tomassetti, Jennifer Lee, Xin Qing

**Affiliations:** ^1^ Division of Hematology and Oncology Harbor‐UCLA Medical Center and The David Geffen School of Medicine at UCLA Los Angeles California USA; ^2^ The Lundquist Research Institute Torrance California USA; ^3^ Department of Pathology Harbor‐UCLA Medical Center and The David Geffen School of Medicine at UCLA Los Angeles California USA

**Keywords:** asciminib, blast phase, chronic myelogenous leukemia, T315I mutation

## Abstract

Patients with chronic myelogenous leukemia (CML) harboring the T315I mutation who progress to blast phase CML while on ponatinib may be successfully treated with asciminib monotherapy following induction therapy with cytotoxic chemotherapy.

## Abstract 2

CML patients with the T315I mutation who progress to blast phase on ponatinib represent a challenging clinical scenario. Asciminib has shown efficacy in chronic phase CML patients with the T315I mutation. Asciminib should be considered for CML patients with the T315I mutation who progress to blast phase while on ponatinib.

## 1 INTRODUCTION

CML is a myeloproliferative neoplasm that represents approximately 15%–20% of adult leukemias.^1^ CML was the first leukemia to be characterized by a consistent chromosomal aberration, the Philadelphia (Ph) chromosome. The Ph chromosome is formed by a fusion that results in creation of a balanced genetic translocation, t(9;22)(q34;q11.2).

Initial therapies used to treat CML consisted of radiotherapy and systemic agents such as busulfan, hydroxyurea, and interferon‐alfa (INF‐a).^2^ This approach controlled the signs and symptoms of chronic phase (CP) CML to some extent, but it did not prevent the inevitable progression to a rapidly fatal blast phase CML. Later, allogeneic stem cell transplant became the first treatment to eradicate the Ph‐positive clone within the bone marrow and offer a potential cure. In fact, in the 1990s, CML became one of the most common indications for allogeneic transplant worldwide.^3^, ^4^

The treatment and prognosis of CML took a dramatic turn with the advent of the first small molecule tyrosine kinase inhibitor (TKI), imatinib. Imatinib was the first drug to specifically target and inhibit the BCR‐ABL fusion protein and was approved by the Food and Drug Administration for the treatment of CP CML in 2001. This targeted approach drastically altered the clinical course of CML, improving the overall survival from 4 to 5 years to that near age matched controls.^5^, ^6^

Since the approval of imatinib, there are now 4 tyrosine kinase inhibitors approved by the US FDA for the first‐line treatment of CP CML: imatinib, nilotinib, dasatinib, and bosutinib. Similar to imatinib, these TKIs all target the ATP‐binding pocket of the ABL1 domain. Clinical trials with second generation TKIs have shown faster and deeper responses and decreased progression to BP CML, but have failed to show improvements in overall survival.^7^, ^8^, ^9^, ^10^ Patients who progress on first‐line TKI are usually given a second‐generation TKI.

Resistance to TKIs is thought to be due to point mutations in the ATP‐binding domain. In particular, the ABL1 T315I mutation, known as the “gatekeeper” mutation, confers resistance to all TKIs with the exception of ponatinib.^9^, ^11^ This T315I mutation comprises about 10%–27% of mutations found in those who fail imatinib and 3%–15% of patients overall.^12^ Until recently, for patients with the ABL1 T315I mutation who fail ponatinib, there were few options outside of allogeneic transplant.^13^ However, with the development of asciminib, patients with the T315I mutation now have an alternative option.

Asciminib is a first‐in‐class STAMP (Specifically Targeting the ABL Myristoyl Pocket) inhibitor. It is an allosteric inhibitor that binds to the myristoyl pocket of the BCR‐ABL1 protein to induce a conformational change and lock the BCR‐ABL protein in an inactive confirmation.^14^ This mechanism is distinct from the previously approved TKIs that all target the ATP‐binding site of BCR‐ABL1. Asciminib targets both native and mutated BCR‐ABL1, including the T315 mutant.^9^ Asciminib was shown to be effective in the phase 1, CABL001X2101 (NCT02081378), dose escalation study of 141 patients with CP CML and nine patients with AP CML who were previously intolerant to or resistant to greater than or equal to 2 prior TKIs who were treated with asciminib monotherapy.^9^ Overall, 48% of all patients had a major molecular response (MMR) by 12 months, including 57% who were previously resistant to or intolerant to ponatinib.^9^ Asciminib was well tolerated with the most common side effects (occurring in 25% or more) being fatigue, headache, and increased lipase.^9^

These promising results prompted the ASCEMBL trial (NCT03106779), a phase 3, randomized, open‐label trial of 233 CP CML patients previously treated with at least 2 TKIs who were given asciminib 40 mg orally twice daily or bosutinib 500 mg orally daily.^15^ In this trial, 25% of those treated with asciminib and 13% of those treated with bosutinib achieved MMR at 24 weeks.^15^ Based on the results of these two trials, in 2021 the US FDA granted accelerated approval of asciminib for the treatment of Ph positive CP CML in adults previously treated with two or more TKIs or with the T315I mutation.

Since the introduction of imatinib and other small molecule TKIs, the rate of progression to blast phase has decreased from 20% to around 1%–1.5% per year.^16^, ^17^ Treatment options for BP CML remain limited and most recommend TKI with or without cytotoxic chemotherapy to achieve a second chronic phase CML followed by allogeneic transplant.^18^ Prognosis is poor with an overall survival of about 8 months without allogeneic transplant.^19^

For patients who are found to have the T315I mutation upon progression to blast phase, ponatinib with or without cytotoxic chemotherapy is a viable option.^20^ However, at this time, there is no FDA‐approved therapy outside of cytotoxic chemotherapy for patients with the T315I mutation who progress to blast phase CML while already on ponatinib. Given its demonstrated efficacy in CP CML patients with the T315I mutation, asciminib with or without cytotoxic chemotherapy may be an effective treatment in this patient population. Here, we present a unique case of a patient with AP CML and the T315I mutation who progressed to myeloid BP CML while on ponatinib and was treated with asciminib.

## 2 CASE PRESENTATION

In July of 2018, a 62‐year‐old woman with no prior medical history presented to an outside hospital for gout where she was told her white blood cell count (WBC) was “very high” and her red blood cells (RBC) and platelets (plt) were “low.” She received a bone marrow biopsy, but never followed up afterwards. Six months later, she presented again to the outside hospital for shortness of breath and was found to have a pleural effusion. At that time, she had a WBC count of 354 K/cumm, plt count of 933 K/cumm, and was reportedly anemic. The differential and blast count at that time is unknown. She received several thoracenteses over the hospital course and was started on hydroxyurea. She was diagnosed with AP CML and was started on imatinib. Upon discharge at the outside institution, she had a WBC count of 144 k/cumm with 6% blasts, hemoglobin of 7.5 g/dl, and plt count of 1184 K/cumm.

After taking imatinib for about 1 week, she presented to our institution with shortness of breath and back pain. Upon presentation, she was noted to have decreased breath sounds in the left lower lung field, splenomegaly, and bilateral lower extremity pitting edema. At that time, her WBC count had decreased to 79.4 K/cumm, hemoglobin of 6.6 g/dl, and plt count of 895 K/cumm. The differential showed 72% neutrophils, 4% bands, 6% lymphocytes, 2% monocytes, 3% eosinophils, 5% basophils, 4% myelocytes, and 4% blasts. Peripheral blood polymerase chain reaction (PCR) for BCR‐ABL1/ABL1 was 57.940% and detected the P210 BCR‐ABL1 fusion transcript. She was continued on imatinib 600 mg per day for AP CML. After 1 month, her WBC count normalized at 5.1 K/cumm, but she remained anemic (hemoglobin 7.8 g/dl) and with thrombocytosis (plt 846 K/cumm). After 6 months of imatinib therapy, her PCR BCR‐ABL1/ABL1 remained persistently elevated at 38.728%. ABL mutational testing was negative, and she was switched to dasatinib 100 mg daily.

After 3 months of dasatinib, her BCR‐ABL1/ABL1 decreased to 12.567%; however she required several medications pauses and dose reductions due to episodes of grade 3‐4 neutropenia. Her BCR‐ABL1/ABL1 nadired at 10.701%, but after about 15 months was again elevated at 94.141%. ABL mutational testing done at that time showed a 944C➔T(T315I) mutation and she was switched to ponatinib.

Ponatinib therapy was also complicated by cytopenias requiring several pauses and dose reductions. After about 3 months, her peripheral blood showed a WBC count of 54.8 K/cumm with 68% basophils. No blasts were seen. Bone marrow biopsy subsequently confirmed AP CML. It was thought that the progression to accelerated phase CML was likely the result of the numerous drug holidays due to cytopenias, and the patient was continued on ponatinib 45 mg daily.

**FIGURE 1:**
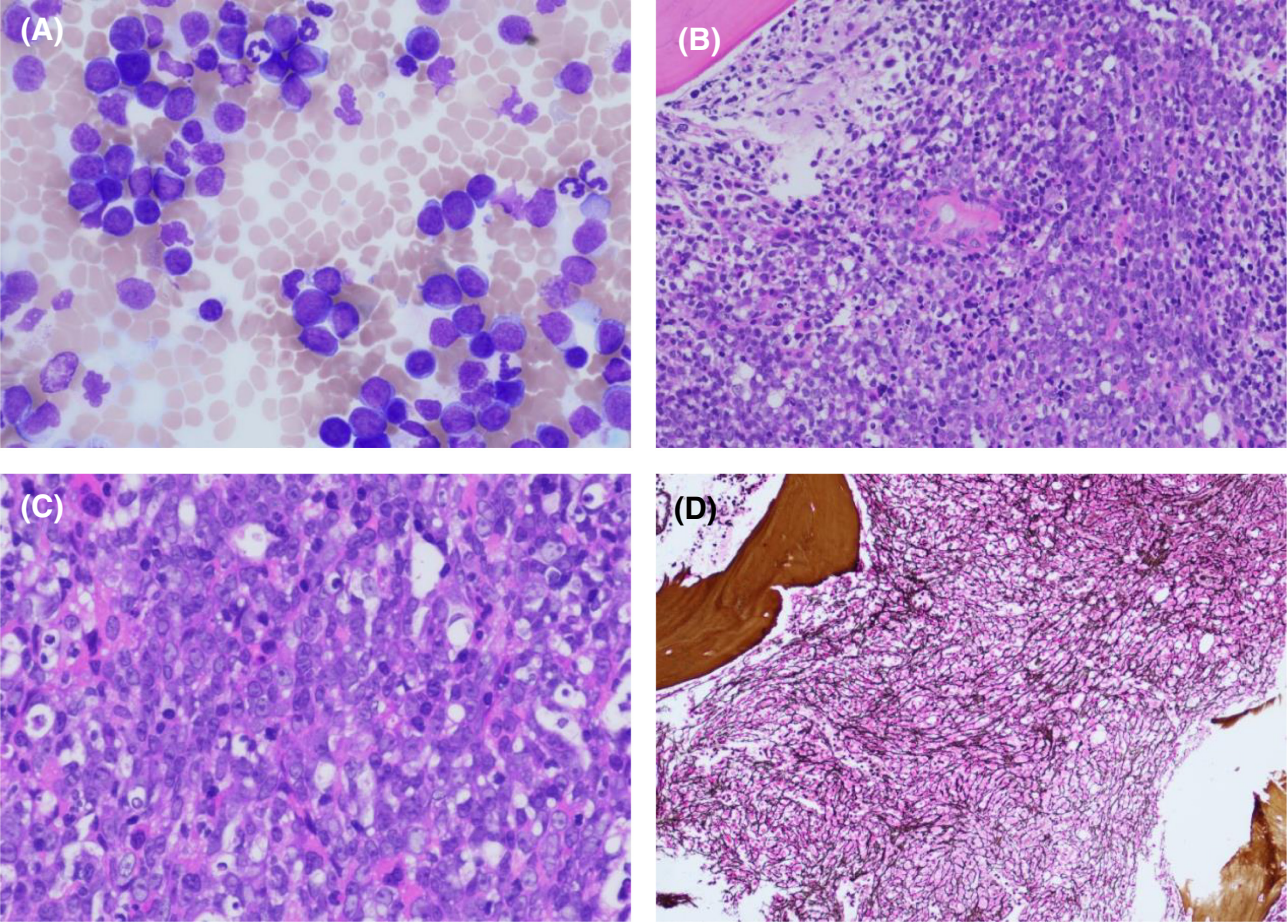
Bone marrow aspiration and biopsy show chronic myeloid leukemia, myeloid blast phase. (A). Bone marrow aspirate smear contains many blasts. (Wright‐Giema stain, original magnification, × 400). (B, C). Bone marrow biopsy section shows sheets of blasts, representing about 70% of bone marrow cellularity. [H&E stain, original magnification, × 200 (B), × 400 (C)]. (D). Myelofibrosis is evident in silver‐stained bone marrow biopsy section. (Original magnification, × 100).

Her counts initially improved; however, 8 months after starting ponatinib, she was found to have a WBC of 35.9 K/cumm and 45% blasts in her peripheral blood confirming progression to BP CML. Bone marrow biopsy showed myeloid blasts harboring the Ph chromosome and the following karyotype: 46,XX,t(7;17)(p15;q23), t(9;22)(9q34;q11.2) (Figure 1). Next‐generation sequencing (NGS) (by Tempus) showed persistent ABL1 T315I mutation, as well as a BCOR R1217F frameshift mutation.

**FIGURE 2:**
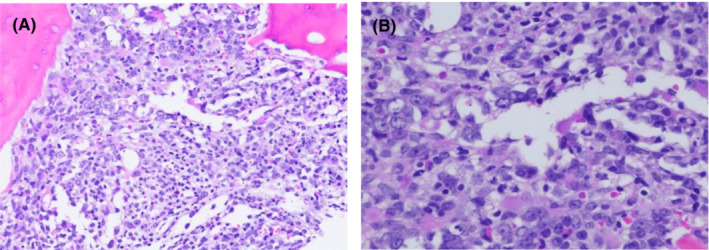
Bone marrow biopsy section shows 5%–10% residual blasts after induction chemotherapy with cytarabine and anthracycline. [H&E stain, original magnification, × 200 (A), × 400 (B)].

She was admitted to our hospital and induced with cytarabine and anthracycline. She underwent a Day 17 bone marrow (at which time her peripheral blood WBC 3.9 k/cumm, Hb 6.7 g/dl, and plt 5 K/cumm), which showed at least AP CML with 5%–10% blasts and focal marked marrow fibrosis (Figure 2). Within a few days, her peripheral blood showed increasing WBC count to 52.3 K/cumm with a blast count fluctuating between 2% and 14%. On Day 19, she underwent re‐induction with venetoclax, fludarabine, and cytarabine. Her course was complicated by neutropenia and various infections including aspergillus pneumonia and COVID‐19 infection. Bone marrow biopsy was not done on Day 28 due to lack of count recovery.

**FIGURE 3:**
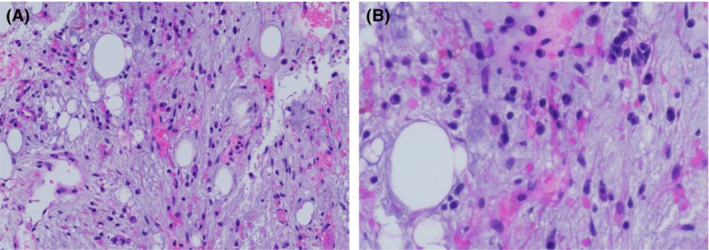
Bone marrow biopsy section shows no residual blasts 6 weeks after starting asiminib. [H&E stain, original magnification, × 200 (A), × 400 (B)].

At 6 weeks post induction, her BCR‐ABL1/ABL1 remained at 92.216% in the setting of persistent pancytopenia (WBC 0.9 k/cumm, Hb 8.1 g/dl, plt 11 K/cumm) without blasts. Given lack of molecular remission and the fact that she had progressed to blast crisis while on ponatinib, asciminib was requested and approved as part of the Managed Access Program (MAP) by Novartis. She was subsequently started on asciminib 200 mg oral twice daily, the approved dosing for patients with ABL T315I mutations in CP CML. Repeat bone marrow biopsy at 3 months post induction therapy (6 weeks after starting asciminib) showed complete response with no blasts and negative minimal residual disease by flow cytometry (Figure 3). Unfortunately, no BCR‐ABL1/ABL1% was done at this time due to logistical reasons. She was referred for allogeneic stem cell transplant.

## 3 DISCUSSION

To our knowledge, this is the first case reported of a patient with CP/AP CML and the ABL1 T315I mutation who progressed to myeloid BP CML while on ponatinib and achieved a CR with MRD negativity after induction chemotherapy followed by asciminib maintenance.

It is well known that progression to blast phase is a result of continued, constitutively active BCR‐ABL activity leading to genetic instability, consequential DNA damage, and impaired DNA repair. This is supported by the fact that reduction in BCR‐ABL by TKI inhibition has been shown to lead to a lower incidence of progression to BP CML.^17^ Consequently, upon progression to BP, most patients show additional chromosomal aberrations. High‐risk chromosomal aberrations associated with short survival in BP CML have been well characterized and include +8, +Ph, i(17q), +17, +19, +21, 3q26.2, 11q23, −7/7q abnormalities, and complex cytogenetics.^21^

Consistent with this, our patient was found to harbor a new t(7;17) in addition a mutation in BCOR on NGS. To our knowledge, the t(7;17) has only been reported once in the literature and was in association with de novo or therapy‐related myelodysplastic syndromes or acute non‐lymphocytic leukemia.^22^ BCOR is located on the X chromosome and is involved in chromatin modification and transcription. It has been reported in about 15% of patients upon progression to BP CML and has been reported to be more common in TKI‐naive patients.^23^, ^24^, ^25^ In our patient, it is unknown whether the BCOR mutation was present prior to BP CML as NGS was only sent at the time of BC diagnosis.

The response to TKI inhibition alone in BP is usually transient, demonstrating that while most cells continue to be sensitive to BCR‐ABL inhibition, there is a component of BCR‐ABL independent growth advantage. In our case, the patient did not receive TKI therapy during AML induction therapy due to the presence of the T315I mutation with prior progression on ponatinib and lack of FDA‐approved therapy. On induction therapy alone, she was not able to achieve a complete remission and her BCR‐ABL1/ABL1 remained above 90%. It was not until she started asciminib that she attained a complete response on bone marrow examination with MRD negativity. Unfortunately, due to logistical reasons, we were unable to also attain a BCR‐ABL1/ABL1 at this time. However, pathology did not note any chronic myeloid leukemia cells present.

To the best of our knowledge, there are no published studies of BP CML patients treated with asciminib. Additionally, there are only limited studies of asciminib in CML patients with the T315I mutation. In the phase 1, CABL001X2101 (NCT02081378) previously mentioned, 28 CP and 5 AP CML patients with T315I mutation were included.^9^ Among all patients with the T315I mutation, 88% achieved a complete hematologic response by 12 months. A MMR was achieved in 25% and 11% of those with CP CML and AP CML, respectively.^9^ In those with CP CML and the T315I mutation who were deemed to have resistance to ponatinib, 20% had a MMR at 12 weeks.^9^ Unfortunately, the phase 3, ASCEMBL trial excluded patients with the T315I mutation.^15^

Since then, a real‐world study of 31 heavily treated CP CML patients also treated with asciminib under the Novartis MAP program was published. In this study, 28 patients (90%) had received three or more prior TKIs including 11 (35%) who had received ponatinib. Of these, 22 patients had intolerance to and 9 patients had resistance to prior TKI.^26^ Of the patients without a baseline complete cytogenetic response (CCyR) or MMR, 48% (8/17) and 33% (8/24) achieved CCyR and MMR, respectively. Of the patients previously treated with ponatinib, 27% (3/11) achieved at least MMR.^26^ Only one patient in this study had a T315I mutation and was treated with asciminib 200 mg orally twice daily. Unfortunately, this patient lost hematological response after 3 months of asciminib treatment and discontinued therapy. Interestingly, in this study, three patients (9.7%) developed grade 4 thrombocytopenia with two of them developing a concurrent grade 4 neutropenia. Like our patient, all of these patients had similar toxicity with previous TKIs.

## 4 CONCLUSION

This case report shows that asciminib, in combination with cytotoxic chemotherapy, may be an effective treatment for CML patients with the T315I mutation who progress to BP CML while on ponatinib. Consistent with prior studies, it also shows that hematologic toxicity, when present, seems to occur across multiple TKIs, in addition to, asciminib.

## AUTHOR CONTRIBUTIONS

S.T. drafted the manuscript. J.L. and X.Q. helped to draft the manuscript and proofread the paper. X.Q. provided the initial diagnosis and pathology images. S.T. and J.L. participated in the clinical treatment. All of the authors read and approved the final version of the manuscript.

## CONFLICT OF INTEREST

Sarah Tomassetti receives research funding from Novartis Pharmaceuticals. The other authors have no conflicts of interests to declare.

## CONSENT

Written informed consent was obtained from the patient to publish this report in accordance with the journal's patient consent policy.

## Data Availability

The data that support the findings of this study are available from the corresponding author upon reasonable request.
